# Impact of surgical intervention on progression to end-stage renal disease in patients with posterior urethral valve

**DOI:** 10.1186/s12301-021-00261-8

**Published:** 2021-12-11

**Authors:** Rishikesh Velhal, Aadhar Jain, Anveshi Nayan, Sujata Patwardhan, Bhushan Patil

**Affiliations:** 1 Department of Urology, Seth GS Medical College and KEM Hospital, Affiliated to Maharashtra University of Health Sciences, Mumbai, India; 2 Seth GS Medical College and KEM Hospital, Affiliated to Maharashtra University of Health Sciences, Mumbai, India

**Keywords:** Posterior urethral valves, Outcome, CKD, ESRD, Urinary bladder neck obstruction

## Abstract

**Background:**

Posterior urethral valve patients present with varied presentations at any age of life and have significant associated morbidity and require long-term follow-up and care.

**Methods:**

This was a single-center ambispective cohort study carried out over a period of 2 years. Patient data regarding the symptoms, investigations, interventions, secondary complications were recorded and were followed up regularly during the study till either normalization of their creatinine level which was maintained up to one-year post-fulguration (non-CKD) or progression to end-stage renal disease (ESRD) requiring renal transplant. Various clinical factors were then compared between these groups.

**Results:**

The age of presentation varies from 6 months antenatal period to a maximum of 34 years. Most common symptom was of lower urinary tract obstruction, followed by recurrent febrile UTI. The interval between disease presentation detection and PU valve fulguration ranged from 6 days to more than 5 years, median duration being 1 month. 85.7% patients had hydroureteronephrosis on initial USG. In VCUG, there was no significant difference found between the presence of reflux and poor renal outcome. Age of presentation greater than 2 years was seen in 52% of patients with CKD compared to only 10% patients in non-CKD group (significant, *p* value 0.02). Among patients who developed CKD, 60% of patients had PU valve fulguration after one month of disease presentation, while in contrast, among the non-CKD group, 80% of patients had it done within one month of disease presentation. (significant, *p* value 0.03).

**Conclusions:**

Late age of presentation, delayed fulguration with high initial creatinine, and failure of serum creatinine to return to normal after one-month post-fulguration are important risk factors in the progression of the disease to ESRD. Symptomatic improvement after interventions does not correlate with progression to ESRD. The number of interventions also does not predict progression to ESRD. Interventions should be chosen wisely on case to restore near-normal physiology and delay progression to ESRD.

## Background

Posterior urethral valves (PUV) result from a congenital malformation of the male urethra at the junction of the membranous and penile urethra that causes obstruction to urinary flow [1]. It is estimated to affect 1:5000–1:8000 male births [2]. PUV patients have a spectrum of clinical presentations. Patients with severe obstruction may present early in the neonatal period with oligohydramnios: intra-uterine growth retardation, pulmonary hypoplasia, and renal dysfunction with hydronephrosis and palpable bladder. Milder forms of obstruction present later, usually with urinary tract infection (UTI), poor urinary stream or urinary incontinence, growth retardation, or with associated kidney failure. Long-term renal outcome remains poor, with as many as 30% of patients progressing to renal failure [3]. Somatic growth in children with PUV may be affected by many factors such as impaired nutrition, renal dysfunction, salt wasting, VUR, and recurrent surgeries.

The current surgical treatment of PUV is primary valve ablation via trans-urethral cystoscopy. If primary ablation is not possible, temporary urinary diversion is achieved by vesicostomy, ureterostomy, or nephrostomy, with valve ablation done later when the urethra can accommodate the resectoscope. Antenatally diagnosed patients can be treated with fetal surgeries such as vesico-amniotic shunting or cystoscopy and valve ablation.

Thus, posterior urethral valve patients present with varied presentations at any age of life and have significant associated morbidity and require long-term follow-up and care.

These patients require various types of surgical interventions to stabilize the disease and to prevent further disease progression and renal function deterioration. It has been observed that a large majority of children with PUV have poor outcomes of medical and surgical management. There is also doubt whether these interventions do really help in halting the disease process or not? With every intervention, patients are subjected to complications and morbidity associated with it.

This study was undertaken with the aims and objective of studying the role of different surgical interventions in management of PUV, the effect of these interventions on symptom modifications and complications arising from them.

## Methods

This was an ambispective, single-center study carried out over a period from Aug 2015 to Nov 2017 after taking institutional ethics committee approval. Convenience sampling was done in which all newly diagnosed patients of PUV during the study period plus all the previous patients of PUV following up in Urology OPD in a tertiary care center were taken in the study after obtaining informed written consent.

Data of patients with PUV were collected from previous and current records regarding age at presentation, their initial symptoms and investigations such as serum creatinine, electrolyte, hemogram, and urine routine microscopy, culture sensitivity, and radiological imaging like ultrasonography, voiding cystourethrography, and functional renal scan. During the duration of the study, patients were followed up for their disease progression by:

1. 3-monthly physical examination, serum creatinine level, and urine routine microscopy.
2. 6 monthly ultrasonography to look for the status of upper tracts and bladder.
3. Urodynamic studies were carried out in every patient one month after PU valve fulguration. Urodynamic studies are not routinely done for patients and 14 patients who were referred from elsewhere did not have this done. However, they have been included in the study.

Data regarding the symptoms, diagnostic imaging, urodynamic studies, time of interventions like PU valve fulguration or other surgical procedures and medical management appropriate for their disease state, the trend of serum creatinine levels following interventions, secondary complications, and associated interventions like percutaneous nephrostomy tube insertion, etc., were recorded in chronological order. In older patients who were following with us or referred from outside, data regarding the same were obtained from their previous medical records. Patients were followed up till either normalization of their creatinine level within the normal range for age which was maintained up to one-year post-fulguration (non-CKD) or progression to end-stage renal disease (ESRD) requiring renal transplant. There was also an intermediate group of patients who progressed to chronic kidney disease (CKD), but their creatinine level reached to certain nadir level, but they did not progress to ESRD during the follow-up period. Various clinical factors were then compared between these groups. The data were analyzed using SPSS version 21. Descriptive analysis of patient characteristics and their management modalities has been done. Quantitative data have been expressed in the form of mean ± 2 SD and qualitative form with median with inter-quartile range. Chi-square was used to check if the difference between the groups having different risk factors for development of CKD was significant or not. RR used to calculate association between trend of serum creatinine and progress to CKD.

## Results

Thirty-five patients of having PUV were included in the study. Their distribution with respect to the age of presentation/detection and serum creatinine levels is shown in Table 1.

**Table 1:** Characteristics of sample

Age at presentation	Number of patients (Total = 35)
ANC	4
0–6 months	9
6–12 months	3
1–2 years	11
2–5 years	2
> 5 years	6

Serum creatinine at presentation (mg%)	Number of patients
0–1	10
01-Feb	19
02-May	3
> 5	3

Ultrasonography features at presentation	Number of patients
Thick trabeculated bladder	15 (42.8%)
Dilated posterior urethra	03 (8.5%)
Normal kidneys	04 (11.4%)
Hydroureteronephrosis	20 (57.1%)
Renal cystic changes	02 (5.7%)
Pyonephrosis	02 (5.7%)
Shrunken kidneys	01 (2.8%)
CMD loss	10 (28.5%)
Raised Echoes	11 (31.4%)

Age at PUV fulguration	Number of patients
0–1 month	5
1–12 months	6
1–2 years	10
2–5 years	5
> 5 years	9

Interval between diagnosis of PUV and Valve fulguration	Number of patients
0–1 month	20
1–12 months	10
1–2 years	2
2–5 years	2
> 5 years	1

All the patients had symptoms of lower urinary tract obstructions in form of either poor flow, straining, urinary retention with or without overflow incontinence, or nocturnal enuresis. Thirty-one patients (88.5%) had a history of recurrent urinary tract infection, eight patients (22.8%) had abdominal distension either due to urinary retention or associated renal rickets, and 13 patients (37.1%) had failure to thrive due to associated metabolic abnormalities. Nine patients (25.7%) had manifestations of rickets at the initial presentation.

Three patients underwent vesicostomy as the initial intervention elsewhere due to the non-availability of a pediatric cystoscope. Rest of the patients underwent PU valve fulguration directly after an initial period of per urethral catheterization. However, ultimately all the patients underwent PU valve fulguration.

Age at PU valve fulguration varied from 6 days of the postnatal period till 34 years of age (Table 1). The interval between disease presentation/detection and valve fulguration is shown in Table 1. 57% of patients had PUV fulguration done within one month of their disease presentation. The reason for this variable time-period is that patients sought medical attention only after onset of severe symptoms even though the disease had been diagnosed earlier radiographically. Twenty-four patients (68.5%) required valve fulguration only once, seven patients (20%) required residual valve fulguration on check cystoscopy after one month, while four patients (11.4%) had valve fulguration thrice due to persistent residual valve.

Initial ultrasonography (USG) reports of patients are shown in Table 1. In voiding cystourethrography (VCUG), 20 patients (57.1%) had reflux ranging from grade III to grade V, 9 patients (25.7%) among them had bilateral reflux. Bladder abnormality such as thick-walled trabeculated bladder with or without diverticuli was present in 18 patients (51.4%) and dilated posterior urethra in 20 patients (57.1%). There was no correlation between the functional status of bladder and development of CKD and ESRD.

All patients were divided into two groups depending upon the creatinine level trend (increasing/decreasing) after 1-month post-initial intervention. Table 2 depicts the number of patients in these groups along with their initial serum creatinine levels and progression to CKD.

**Table 2:** Factors affecting CKD

Relation between post-fulguration creatinine level trend and progression to CKD			
	CKD	Non-CKD	Total number of patients
Increasing creatinine	14 (87.5%)	2 (12.5%)	16
Decreasing creatinine	11 (57.9%)	8 (12.1%)	19
Total	25	10	35

Relation between age of presentation and CKD			
Age at presentation	CKD (25)	NON-CKD (10)	
ANC	3	1	Significant difference in progression to CKD exists between age of presentation less than or equal to 2 years and that > 2 years with *p* < 0.05
0–1 month	3	1	
1–12 months	5	1	
13–24 months	1	6	
2–5 years	6	1	
> 5 years	7	0	

Relation between time interval to fulguration and CKD progression			
Interval between disease presentation and fulguration	CKD (25)	Non- CKD (10)	
< 1 month	10	8	
1–12 months	10	1	Significant difference in progression to CKD exists between patients who had valve fulguration within 1 month of presentation versus those after 1 month with *p* < 0.05
1–2 years	3	0	
2–5 years	1	1	
> 5 years	1	0	

We further divided the patients depending upon the number of surgical interventions required during their disease course. In the first group of fifteen patients (42.8%), only PU valve fulguration required. Out of these 15 patients, only six patients (17.1%) required re-fulguration. In the other group (Multiple Intervention group) of 20 patients (57.1%), other surgical interventions were required in addition to valve fulguration such as temporary vesical or supravesical urinary diversions or other auxiliary procedures for bladder management. Among these 20 patients, 15 patients underwent PUV fulguration once, one patient underwent PUV fulguration twice, and four patients required PUV fulguration thrice, besides undergoing other interventions. On urodynamic studies, 14 patients had bladder abnormalities such as low compliance or detrusor instability, out of which eight patients required surgical management. The other six patients managed on anticholinergics and/or CIC.

**Fig. 1:**
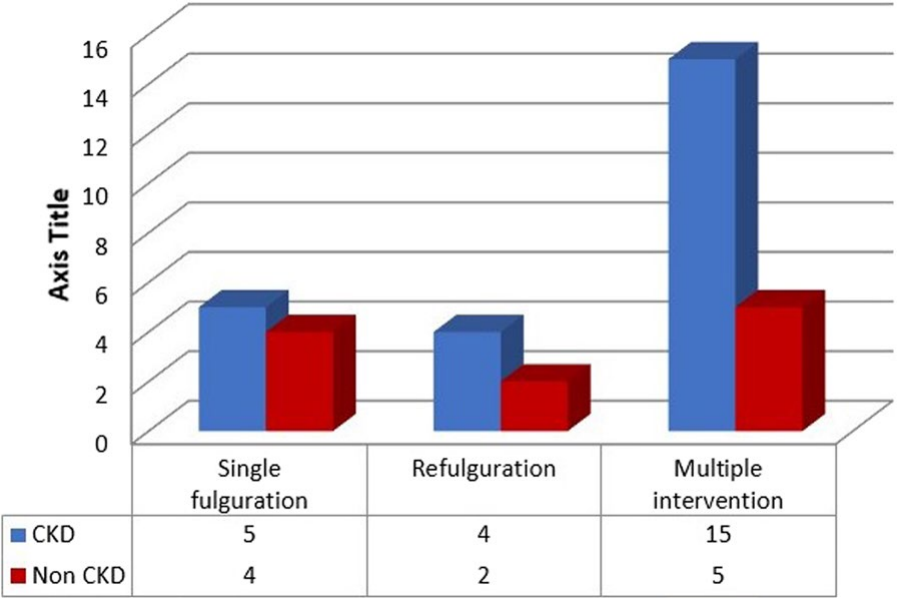
Relation between number of interventions and progression to CKD

In single fulguration group 5 out of 9 patients (55%), in re-fulguration group 4 out of 6 patients (67%) and in multiple interventions group 15 out of 20 patients (75%) progressed to CKD (Fig. 1).

Nineteen patients (54.2%) required urinary diversions in the form of either percutaneous nephrostomy, vesicostomy, ureterostomy, suprapubic cystostomy, or combinations of these for indications like persistent upper tract obstructions, acute renal failure, or infective complications. Among these patients, 13 patients (68.4%) had a decrease in creatinine level post-interventions temporarily out of which 9 patients (47.3%) later progressed to CKD. The other six patients (31.6%) who underwent urinary diversions had persistent or increased creatinine levels and disease progression to CKD.

Analyzing the effects of interventions on symptoms modification and their complications, thirty-one patients (88%) had improvement in their presenting symptoms after initial fulguration/diversion. Nine patients (25.7%) had resolution of obstructive changes on ultrasonography. Seventy-one percent (71%) patients had an episode of febrile UTI post-intervention requiring admission. Fifty-one percent of patients among them had pyelonephritis or pyonephrosis requiring further intervention or diversion. Eight patients (22.8%) developed significant post-surgery complications which prolonged their hospital stay, needed more interventions for managing them, and an overall increase in morbidity. Some other complications encountered in were vesicocutaneous fistula (1 patient), calculus disease in obstructed or diverted system (1 patient), burst abdomen (1 patient), post-cystoplasty intra-abdominal urine leak (1 patient), febrile UTI with acute renal failure (4 patients), stenosis at ureteric reimplant site (1 patient), hematuria with bladder clots in augmented bladder (1 patient).

Finally, comparing between patients who progressed to CKD or ESRD and other patients whose creatinine level remained normal, among the 25 patients who developed CKD, 23 patients (92%) had persistent hydroureteronephrosis post-PUV fulguration. In the non-CKD group, 7 out of 10 patients (70%) either had no obstructive changes or resolution of obstructive changes post-fulguration. 75% of patients requiring multiple interventions progressed to CKD. The relation between the age of presentation and progression to CKD is shown in Table 2. 52% of the CKD group had the age of presentation > 2 years, while in the non-CKD group, only one patient (10%) presented above 2 years of age (statistically significant, *p* value < 0.05). In the CKD group, 60% of patients (15/25) had their valve fulguration after one month of disease presentation, while in the non-CKD group 80%, of patients (8/10) had their fulguration before one month of disease presentation (Table 2). This relation is statistically significant (*p* value < 0.05).

## Discussion

Posterior urethral valve is the most common cause of congenital lower urinary tract obstruction in male children. It is a complex developmental disease whose outcome depends upon the dynamic interplay between lower tract obstructions and upper tract physiology in response to it. The initial injury to the developing urinary system starts in utero, and it continues during the childhood of the patient till they progress to CKD and finally ESRD. Overall, approximately 30% of patients have CKD before adolescence and one-third of them progress to ESRD [4]. Surgical intervention is the mainstay of management in PU valve, and these patients require a variable number of interventions from time to time according to their disease status. But there is still a lack of clarity that as to whether these interventions really help in preventing disease progression or not? Ultimately, the final disease outcome in patients with PUV depends on multiple factors and requires long-term regular follow-up. In our study, we tried to find out the major disease-related factors which determine the final renal outcome, progression to CKD and ESRD in the Indian scenario, and effects of surgical interventions in the management of patients with PUV.

Some studies have reported that the younger the age at presentation the poorer the outcome will be while others believe that late presentation is associated with a higher risk of developing chronic renal insufficiency [5, 6]. We found a statistically significant difference between the age of presentation above 2 years and progression to CKD in our study (52% of CKD vs 10% of non-CKD, *p* value < 0.05). However, we could not demonstrate a significant difference in renal outcome between patients who presented below the age of 1 month or antenatal period and patients above 1 month of age. Delayed presentation in our study could be due to the absence of antenatal ultrasound, lack of awareness, and neglect toward the symptoms in the infantile period and low socioeconomic strata with difficult access to healthcare facilities.

Mean serum creatinine was found to be high in patients who progressed to CKD (2.24 mg% in CKD group vs 1.16 mg% in non-CKD group, *p* value < 0.05). Nickavar et al. in 2008 concluded that initial serum creatinine is a valuable factor for predicting renal outcome in patients having PUV with initial creatinine above 1 mg% having higher risk of progression to ESRD [7]. In our study, due to the small sample size and skewed data, mean creatinine was found to be on the higher side as compared to others.

In this study, lower urinary tract symptoms were the most common symptom presentation which is consistent with other studies. The second most common symptom findings were recurrent febrile UTI (71%) requiring antibiotic course or indoor admission. Approximately 40% of patients were diagnosed as having PUV during their admission for febrile UTI indicating neglect toward the LUTS in children. 17.1% patients presented as acute kidney injury and half of them required dialysis for azotemia. Failure to thrive was seen in almost one-third of patients.

We found out a significant difference in CKD progression and interval between disease presentation and valve fulguration of more than one month (*p *value < 0.05). In the non-CKD group, 80% of patients underwent fulguration within one month of their presentation irrespective of their actual age of presentation. Nawaz et al. found out 78% of patients with residual valve on relook cystoscopy and recommended relook cystoscopy should be routinely done after primary fulguration to detect persistent obstruction [8]. We routinely carry out relook cystoscopy after one month in all patients, and in this study, 31.4% of patients required residual valve fulguration.

On USG, patients with persistent upper tract dilatation after fulguration, raised echoes, loss of corticomedullary differentiation, and renal dysplasia were associated with progression to CKD. In VCUG, there was no significant difference found between the presence of reflux and poor renal outcome. However, persistence of reflux even after fulguration and reflux as the pop-off mechanism was associated with reduced kidney function and progression to CKD. Severe alteration in bladder architecture, diverticuli, sacculations, and reflux on VCUG is found to be associated with hostile bladder on urodynamic studies.

Patients with posterior urethral valves and severe bladder dysfunction in whom nadir creatinine remains increased are at risk for upper urinary tract deterioration, further requiring renal replacement therapy [9]. Coleman et al. suggested that patients with nadir creatinine > 75 micromols/l (0.85 mg/dl) should be considered at high risk for chronic renal insufficiency, while patients with nadir creatinine ≤ 35 micromols/l (0.4 mg/dl) should be considered low risk [10]. Depending upon serum creatinine level post-one-month initial intervention, we divided patients into two groups. In the first group where creatinine increased post-fulguration, 87% of patients progressed to CKD, while in the other group, where creatinine decreased or remained stable, 58% progressed to CKD, though this was not statistically significant. (RR = 1.511).

Apart from PUV fulguration, some patients require other surgical interventions in form of urinary diversions such as vesicostomy, percutaneous nephrostomy, ureterostomy, and auxiliary procedures for bladder management and reconstructive procedures such as Clam/augmentation cystoplsty, Mitrofanoff's procedure, ureteric reimplantation, and nephrectomy. From various studies in past, it had been observed that primary valve fulguration is associated with better bladder function than vesicostomy and should be the treatment of choice in PUV. Also, vesicostomy and ureterostomy have distinctly different effects on bladder function with altered capacity and compliance [11]. Ghanem et al. in 2005 observed that in patients with PUV temporary high diversion of the Sober type does not have a negative influence on bladder function. It immediately releases high intrarenal pressures but only improves renal function temporarily and may contribute to the postponement of the time of end-stage renal failure [12]. Chua et al. after a follow-up period of 15 years concluded that urinary diversion following valve ablation in children with CKD stage 3 associated with PUV may temporarily delay progression to ESRD. However, no long-term benefit was noted from diversion in the ultimate incidence of ESRD, suggesting that these interventions should be considered as a temporizing measure [13]. Ghali et al. suggested that short-term percutaneous nephrostomy drainage before doing supravesical diversion helps in identifying patients requiring diversion. Moreover, high loop ureterostomy is actually associated with more complications than vesicostomy or primary valve ablation and is not implicated in prevention of progression to renal insufficiency [14]. Thus, there is questionable importance of diversion procedures in PUV patients as they are necessary at a particular stage of the disease to tackle acute complications such as UTI, acute kidney injury or to decompress persistently obstructed upper tract system. Seventy-five percent of patients who required multiple interventions later progressed to CKD as compared to 60% of patients who did not require interventions other than fulguration, although this difference was not statistically significant. Nineteen patients required diversions in our series,13 out of 19(68%) patients had improved renal outcome for an initial period, but 9 out of 13 (47%) of these patients later progressed to CKD. In the other 6 patients (31.6%) creatinine level never decreased even after diversion. Thus, our study also supported the fact that these diversion procedures are only temporary measures and their value in preventing disease progression requires additional evidence and long-term follow-up. The major role in the management of PU valve is the preservation of bladder physiology and to avoid the formation of "Valve bladder" in patients progressing to ESRD [15]. Unstable or hostile bladder is troublesome in view of renal transplant as chances of graft failure are there. The literature review has mixed inferences regarding the time of bladder augmentation concerning transplant and their outcomes. Jesus et al. in 2015 noted that augmentation cystoplasty is feasible after renal transplant, with complication rates similar to the ones performed beforehand. Since a considerable number of PUV patients with high-pressure bladders eventually develop myogenic failure, it seems logical to postponing AC in this population, as long as they are under close surveillance [16]. Augmentation cystoplasty increases the risk of UTI after transplant, it should be constructed preemptively only if the risks associated with increased bladder pressures exceed those associated with cystoplasty [16]. In our study, seven patients underwent surgery and one patient underwent botox instillation for unstable low compliant bladder and six patients managed on anticholinergics.

Patients undergoing such complex procedures are also at high risk of developing complications. The most common complication was febrile UTI (71%) requiring admission with 50% of them requiring further intervention/diversion for pyonephrosis/pyelonephritis. 22% had major complications requiring long-term admission increasing their morbidity and financial burden to already exhausted parents. One of the major downsides of the PU valve disease and their interventions is a significant amount of loss of school days of children affecting their educational careers. Forty percent of our patients had dropout of a school-year or more due to their troublesome symptoms and or hospital admissions or indwelling catheters in some. Thus, it is obvious that though these surgical interventions form an essential part in the management of PU valve patients in terms of relief of obstruction, symptom alleviations, and help in some to prevent or delay disease progression, yet they are associated with inevitable complications.

Ultimately three patients in our series progressed to ESRD, one of whom presented at 34 years of age with bilateral small shrunken kidneys awaiting transplant. The second patient presented antenatally but had persistent upper tract changes post-fulguration with renal dysplasia and high creatinine and underwent multiple interventions and finally renal transplant at the age of 9 years. The third patient had delayed presentation after 2 years of age with high creatinine, required multiple interventions, and underwent transplant at the age of 12 years.

*Limitations* Considering the rarity of the disease and longer duration of disease course, establishing a large enough database is difficult. Being conducted in a low-middle income country setting, the presentation of patients is also very heterogenous because patients only seek consultation when they have symptoms which affect their daily life. This has an impact on the statistical analysis. Our follow-up period was short as compared to the natural history of illness because of lost to follow-up of many patients due to the covid-19 pandemic. Thus, longer follow-up duration with a larger sample size and higher number of early age patients required with regular follow-up to further highlight the natural course of the disease and variable factors affecting it. Also, confounding factors like different surgeons and place of performing interventions are present.

## Conclusions

Late age of presentation, delayed fulguration with high initial creatinine, and failure of serum creatinine to return to normal after one-month post-fulguration are important risk factors in the progression of the disease to ESRD. Symptomatic improvement after interventions does not correlate with progression to ESRD. The number of interventions also does not predict progression to ESRD. Patients requiring interventions have a high (23%) postoperative further procedures increasing their morbidity. Although no algorithm or guideline can generalize the management of PU valve patients, these interventions should be chosen wisely on case-to-case basis considering the anatomy and pathology of the affected urinary tract and functional renal status so as to restore near-normal physiology and delay progression to ESRD.

## Data Availability

The data which was collected from patients which was analyzed for the study are available in Department of Urology, Seth GS Medical College and KEM Hospital, Mumbai. Access to this data can be provided with permission from Head of Department of Urology and Dean of Seth GS Medical College and KEM Hospital, Mumbai.

## Abbreviations

PUV: Posterior urethral valves
PU: Posterior urethral
ESRD: End-stage renal disease
UTI: Urinary tract infection
VUR: Vesicouretral reflux
VCUG: Voiding cystourethrogram
CKD: Chronic kidney disease
CMD: Corticomedullary differentiation
AC: Augmentation cystoplasty
CIC: Clean intermittent catheterization
